# Adaptation of the Transcriptome and miRNAome in Response to Hepatocellular Hypoxia

**DOI:** 10.3390/ijms27156886

**Published:** 2026-08-01

**Authors:** Jenica H. Kakadia, Cristiana Iosef, Ilka U. Heinemann, Victor K. M. Han

**Affiliations:** 1 Department of Biochemistry, Schulich School of Medicine and Dentistry, The University of Western Ontario, London, ON N6A 5C1, Canada; 2 Children’s Health Research Institute, London Health Sciences Centre, London, ON N6A 5W9, Canada; 3 Department of Pediatrics, Schulich School of Medicine and Dentistry, The University of Western Ontario, London, ON N6A 5C1, Canada

**Keywords:** hypoxia, microRNAs, glycolysis, cell proliferation

## Abstract

Inadequate supply of oxygen causing hypoxic cellular stress drives metabolic reprograming across diverse physiological conditions, including cancer. The activation of hypoxia-inducible factors (HIFs) to facilitate adaptation to low oxygen environments is well-characterized; however, post-transcriptional regulation by microRNAs (miRNAs) is poorly understood. Here, we investigated the mRNA and miRNA response in hypoxia-mediating reduced growth and cellular metabolism. Next-generation sequencing revealed the impact of hypoxia in cultured human hepatocellular carcinoma cells and identified over 400 mRNAs and 140 miRNAs that were differentially expressed. Hypoxia upregulated mRNA transcripts associated with glycolysis, DNA replication and the PI3K (phosphoinositide 3-kinase)-Akt pathway, which promote anaerobic metabolism for energy production, decreased cell proliferation and genomic instability, respectively. Upregulated miRNAs included miR-197-3p and miR-766-3p, targeting genes involved in lipid biosynthesis and metabolism. Downregulated miRNAs included miR-33a-5p and miR-15a-5p, targeting genes involved in glycolysis and lactate metabolism. Notably, miR-6834 emerged as a potential regulator of mechanistic target of rapamycin (mTOR) signaling and insulin-like growth factor binding protein-1 (IGFBP-1) signaling; miR-6834 overexpression altered 4E-BP1 phosphorylation levels and IGFBP-1 secretion. These findings provide critical insights into miRNA–mRNA regulation of metabolic adaptation and highlight miRNAs as potential targets for interventions with relevance to tumor biology and other hypoxia-associated conditions.

## 1. Introduction

Hypoxia is a fundamental physiological and pathological condition that disrupts cellular homeostasis. The generation of reactive oxygen species (ROS) results in oxidative stress, inflammation, and organ dysfunction [1]. ROS are required for appropriate cell function; however, moderately higher levels of ROS in cancer cells can contribute to tumor production and progression as it induces DNA mutation, cancer cell metabolism changes, tumor metastasis, and tumor drug resistance [2]. The transcriptome of cells subjected to prolonged hypoxia showed increased levels of markers related to quiescence, epithelial–mesenchymal transition (EMT), and chemoresistance [3]. The cancer hypoxic microenvironments are a result of rapid cellular proliferation with inadequate vascularization; adaptive signaling pathways are activated in hypoxia, a process majorly mediated by the transcription factor hypoxia-inducible factor 1 (HIF-1), to promote cell survival and metabolic reprogramming [4]. Transcriptional regulation by HIF-1 mediates angiogenesis and promotes a shift to anaerobic metabolism such as by inducing glycolytic enzymes. The promotion of angiogenesis and anaerobic metabolism by HIF-1 contributes to tumor cell survival [5]. HIF-1 additionally modulates the phosphoinositide 3-kinase (PI3K)–Akt and mechanistic target of rapamycin (mTOR) pathways. mTOR can form two complexes, mTORC1 and mTORC2, which coordinate cellular metabolism, growth, and survival under low oxygen conditions [6]. mTORC1 activity is regulated by a variety of stimuli, including growth factors, amino acids, and energy levels [7]. mTORC2 activity is affected in hypoxia by the PI3K and Akt signaling cascade, and is a major regulator of cell proliferation, survival, and metabolism. AKT activity and phosphorylation was previously shown to be regulated by miRNAs in epithelial ovarian cancer cells [8]. Aberrant mTOR signaling is commonly observed in cancer [9,10] and mTOR inhibitors have been investigated as therapies in clinical trials; however, their effectiveness can be dampened by therapeutic resistance. Additionally, the miRNAs involved in hypoxia to impact mTOR and Akt signaling are not well-known.

While transcriptional responses to hypoxia have been extensively characterized, post-transcriptional regulation, such as by microRNAs (miRNAs), is poorly understood. miRNAs are small, endogenous, non-coding RNAs, which post-transcriptionally regulate gene expression by interacting with 3′ untranslated region (UTR) of their target messenger RNAs (mRNA) to inhibit translation or promote degradation. miRNAs contribute to regulation of genes involved in metabolic programming or cellular growth and differentiation. They play an important role in appropriate development, and have been implicated in processes critical to fetal and placental growth, including trophoblast invasion, placental development, and angiogenesis. Many tissues express and secrete miRNAs for cell–cell communication [11,12]. However, only the role of a few miRNAs is known, while the majority remain unestablished.

HepG2 cells, derived from hepatoblastoma, are a well-established in vitro model for studying hypoxia-driven metabolic adaptation. These cells retain many liver-specific metabolic functions and exhibit robust HIF-1 signaling in hypoxia [13,14], making them particularly suitable for investigating hypoxia-induced changes in gene expression and metabolic regulation. The liver plays a major role in energy metabolism, as it is a primary contributor to absorptive glucose storage. Notably, studies show that secretory miRNA levels in the blood and other body fluids significantly correlate with cancer progression [15]. Thus, studying the altered miRNA expression profile of cells in response to hypoxic conditions may provide insight into metabolic adaptation that are relevant across disease contexts, including cancer. miR-122 is well-studied as it comprises approximately 70% of all hepatic miRNAs [16]; its downregulation leads to increased hepatic fatty-acid oxidation and decreased plasma cholesterol levels and hepatic fatty-acid and cholesterol synthesis [17]. miRNAs are associated with genes related to angiogenesis and cell proliferation [18], processes altered in hypoxia, may contribute to tumorigenesis and tumor progression. miR-210, upregulated by hypoxia-inducible factor 1 (HIF-1) in hypoxia, has downstream targets involved in cell migration and angiogenesis [18,19]. miRNA expression profiles are dysregulated in cancer, implicating miRNAs as potential diagnostic markers and therapeutic targets.

Insulin-like growth factor-1 (IGF-1) plays a key role in fetal growth and development [20], as it is involved in regulating glucose metabolism and promoting cell growth and proliferation. Decreased IGF-1 signaling is found in clinical and animal models of intrauterine growth restriction (IUGR), where the growth rate of a fetus falls below its growth potential [21,22,23]. Elevated circulating IGF-1 levels are directly associated with various cancers, including breast and prostate cancer [24]. The liver is a primary source of IGF-1 and IGF binding protein-1 (IGFBP-1) synthesis [25]. IGFBP-1 sequesters IGF-1, decreasing IGF-1 bioavailability. Phosphorylation of IGFBP-1 at discrete sites (Ser101, Ser119, and/or Ser169) increases its binding affinity for IGF-1 [25,26] and further decreases IGF-1 bioavailability and impairs cellular growth [25,27]; however, the specific kinases regulating these phosphorylation events are not known. The serum levels of IGFBP-1 has been proposed as a biomarker for both cancer and diverse hepatic diseases [28]. IGF-1 receptor (IGF-1R) and other growth receptors can activate PI3K to regulate and promote growth [29]. Hypoxia can significantly inhibit cellular proliferation [30] and results in increased IGFBP -1 phosphorylation, decreasing growth factor signaling [31]. Reduced IGF-1 signaling following prolonged hypoxia is an adaptive response to sustain metabolic demand [32]; however, it happens at the expense of cell growth/proliferation. Therefore, the crosstalk between HIF-1, PI3K/mTOR, and IGF-1 signaling requires a careful balance to prevent adverse growth conditions in either direction in response to hypoxia.

In this study, we determined the role of miRNAs in cellular metabolism and growth during hypoxia in HepG2 cells as an in vitro model for fetal hepatocytes. Hypoxia in HepG2 cells has been shown to induce HIF-1 signaling and downstream targets regulating vascularization, as well as promoting the accumulation of IGF-1R [33]. Here, we examine mRNA and miRNA expression profiles in response to hypoxia, which may be correlated with IUGR and cancer pathophysiology, including alterations in glucose and lipid metabolism and other cellular adaptations. Here, we showed that hypoxia induced differential regulation of mRNA and miRNAs and identified miR-197-3p and miR-766-3p to be upregulated, associated with decreased lipid metabolism, as well as miR-33a-5p and miR-15a-5p to be upregulated, which may increase glycolysis and lactate metabolism. In addition, these miRNAs may also be involved in the regulation of mTOR signaling, metabolism and growth.

## 2. Results

HepG2 cells derived from a well-differentiated hepatocellular carcinoma share key proteome and secretome characteristics with primary human and fetal hepatocytes [13,34]; the HepG2 secretome mirrors the fetal liver [13,14,34,35]. Importantly, HepG2 cells are widely used to study hypoxia-driven changes in hepatocellular carcinoma, such as in EMT [36], and HIF-1-mediated changes in glycolysis, angiogenesis, and resistance to oxidative stress, but little is known about the adaptation of the miRNAome to low oxygen concentrations.

### 2.1. Hypoxia Results in Significantly Changed mRNA and miRNA Profiles, Increases Glycolysis, and Decreases Folate Biosynthesis and DNA Replication

**Figure 1:**
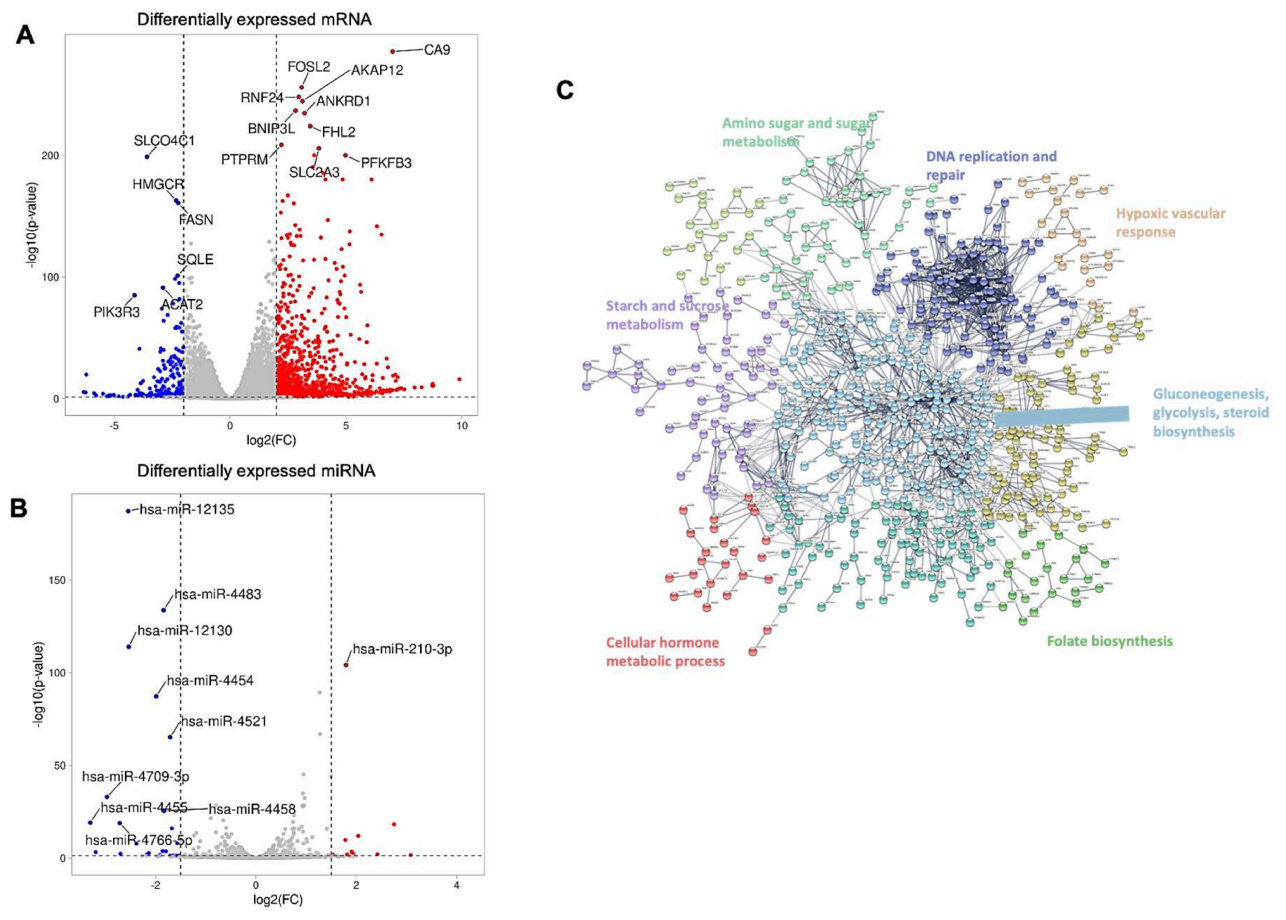
Differentially expressed mRNA and miRNA reveal changes in key functional pathways. Volcano plots of significantly differentially expressed (DE) mRNA (**A**) and miRNA (**B**) in hypoxia compared to normoxia were generated using VolcaNoseR. There are a total of 3529 DE mRNAs and 466 DE miRNAs. All significantly DE genes are represented in terms of their measured expression change (*x*-axis) and the significance of the change (*y*-axis). The significance is represented in terms of the negative log_10_ of the adjusted *p*-value, so that more significant genes are plotted higher on the *y*-axis. The upregulated genes (positive log_2_ fold change) are shown in red, while the downregulated genes (negative log_2_ fold change) are in blue. A significance cut-off of 1.3 (adjusted *p*-value <0.05) was applied. Fold change thresholds were 2-fold for mRNA and 1.5-fold for miRNA. A Search Tool for the Retrieval of Interacting Genes/Proteins (STRING) functional protein association network was created to visualize DE mRNA with >2-fold change (**C**) in hypoxia compared to normoxia. Computational prediction shows direct (physical) and indirect (functional) associations to predict the role of altered mRNA transcript levels. Functional groups are color-coordinated and labeled.

To identify transcriptomic changes and their potential regulation by miRNAs, we utilized concurrent mRNA and miRNA sequencing to identify cellular metabolic and growth pathways associated with hypoxia. Principal component analysis (Figure S1) shows separation of our two treatment conditions, HepG2 cells cultured in either standard culture conditions (normoxia) or with 1% oxygen (hypoxia) for 24 h. We generated volcano plots using VolcaNoseR [37] for differentially expressed (DE) mRNA and miRNAs (Figure 1A,B) in hypoxia compared to normoxia. We applied a 2-fold change cut-off for mRNA analyses, and 1.5-fold change cut-off for miRNA analyses and a significance cut-off of adjusted *p*-value <0.05. Of the 20,241 total mRNAs detected (Table S3), we found that in hypoxia compared to normoxia, 1423 mRNAs were significantly decreased (Table S4) and 2667 mRNAs were significantly increased (Table S5) in abundance by 2-fold. Of the 1365 total miRNAs detected, 63 miRNAs were significantly decreased (Table S6) and 80 miRNAs were significantly increased (Table S7) by 1.5-fold. Significantly and differentially expressed mRNAs were grouped using STRING analysis software [38,39] (Figure 1C), and revealed multiple functional pathways significantly changed in hypoxia.

In hypoxia, we found 24 significantly increased DE transcripts of genes associated with glycolysis and gluconeogenesis. Hexokinase 1 (HK1), which phosphorylates glucose to produce glucose-6-phosphate as the first step of glycolysis, was greatly increased (+30-fold, *p* < 0.001). In hypoxia, decreased ability to undergo oxidative phosphorylation has been shown to increase glycolytic genes, mediated in part by HIF-1 [40,41]. Many transcripts encoding additional glycolysis genes were strikingly increased in hypoxia, including hexokinase 2 (+5.3-fold, *p* < 0.001) and phosphofructokinases PFKFB3 (+31-fold, *p* < 0.001) and PFKFB4 (+8.0-fold, *p* < 0.001). Additionally, we found concurrent changes in miRNAs that may regulate glycolysis and gluconeogenesis. Levels of miR-15a decreased in hypoxia (−1.6-fold, *p* < 0.001); miR-15a has been shown to regulate aldolase fructose-bisphosphate A (ALDOA, +2.7-fold, *p* < 0.001) [42]. Thus, congruent with literature [43], hypoxia promoted a shift toward glycolytic ATP production in HepG2 cells, characterized by increased expression of canonical regulators of glucose uptake and glycolytic flux.

Transcripts most decreased in abundance were associated with DNA replication (Figure 1C), which may impact cell division and growth. Key downregulated genes included members of the DNA polymerase family, DNA polymerase epsilon 2 (POLE2, −2.7-fold, *p* < 0.001) and 3 (POLE3, −2.6-fold, *p* < 0.001) and DNA polymerase alpha 2 (POLA2, −2.4-fold, *p* < 0.001). In mice, disruption of these genes leads to embryonic lethality and abnormal development [44]. Elevated POLE2 levels are correlated with colorectal cancer (CRC) progression by promoting EMT; POLE2 silencing dramatically CRC cell aggressiveness by decreasing proliferation, migration, and invasion [45]. Additionally, proliferating cell nuclear antigen (PCNA), a key factor in both DNA replication and cell cycle regulation, had decreased mRNA levels (−2.3-fold, *p* < 0.001). miR-21 levels were increased in hypoxia (+1.9-fold, *p* < 0.001), while its targets [46], mini-chromosome maintenance complex component 2 (MCM2, −2.1-fold, *p* < 0.001) and 4 (MCM4, −2.2-fold, *p* < 0.001) had decreased mRNA levels.

In hypoxic conditions, we also observed a downregulation of genes involved in folate biosynthesis (Figure 1C), a critical process in DNA synthesis and methylation. Thus, this altered signaling may exacerbate the impairment of DNA replication in hypoxia. Folate is crucial for de novo synthesis of nucleosides adenosine and guanosine [47] as well as DNA stability, and its decreased biosynthesis can impact cellular growth and proliferation. Genes encoding key enzymes in the folate biosynthesis pathway, such as dihydrofolate reductase (DHFR, −2.1-fold, *p* < 0.001) and phenylalanine hydroxylase (PAH, −2.7-fold, *p* < 0.001) had decreased mRNA levels in hypoxia. miR-21 levels, which can impact folate-related genes such as DHFR [48], was increased in hypoxia. Consequently, reduced DNA replication efficiency and decreased folate biosynthesis may contribute to poor fetal development by limiting cellular growth and proliferation. Folate deficiency is associated with adverse pregnancy outcomes, and epidemiological studies correlate decreased folate intake with increased risk of lung, oropharyngeal, esophageal, stomach, colorectal, prostate, and breast cancer [49].

This data demonstrates that during hypoxia, anaerobic glycolysis is increased to compensate for poor oxygen availability, while folate biosynthesis and DNA replication were decreased.

### 2.2. The PI3K-Akt Signaling Pathway Is Involved in the Hypoxic Response

**Figure 2:**
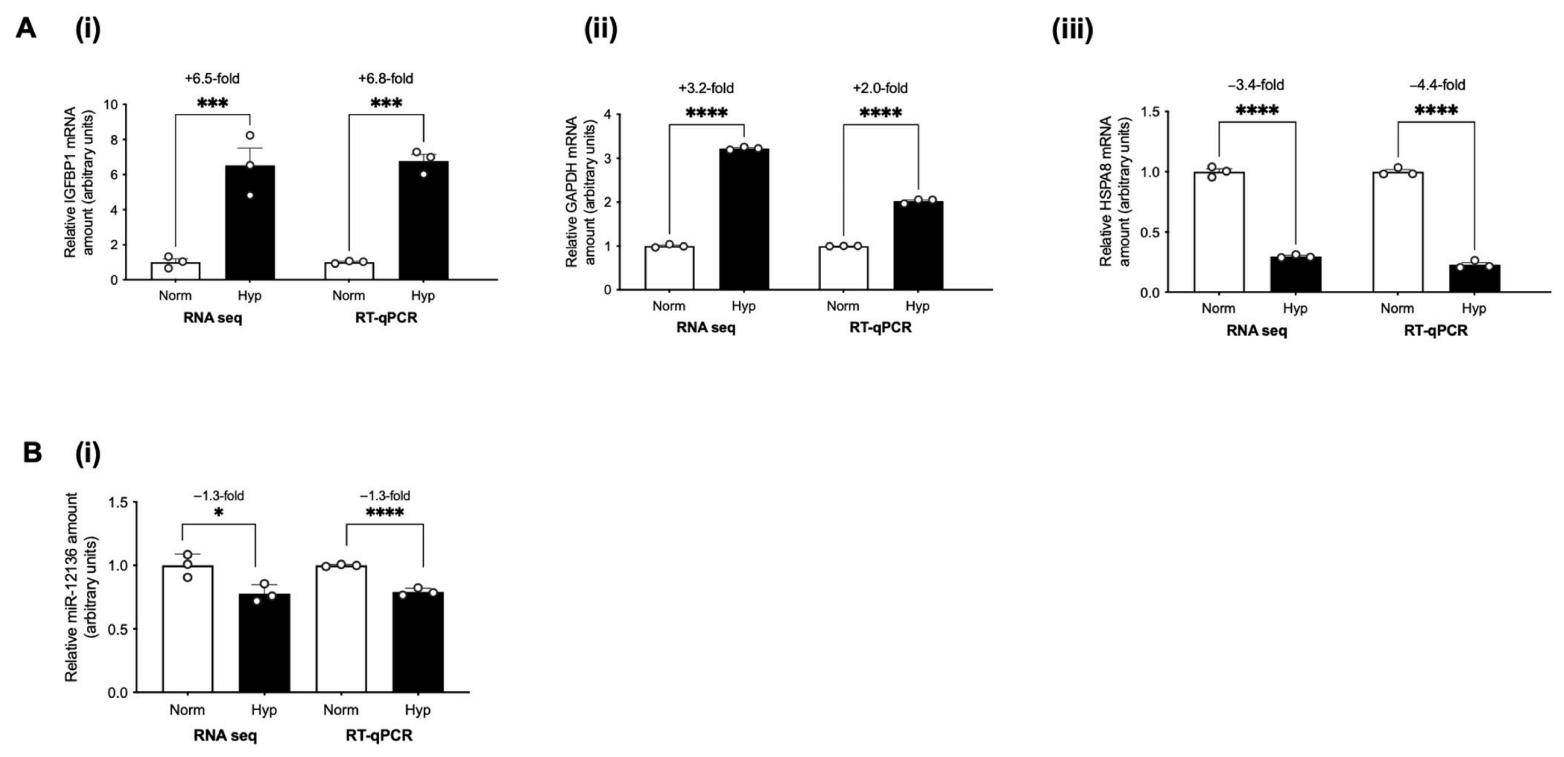
RT-qPCR validation of RNA sequencing data. RT-qPCR validation of RNA sequencing data was performed following the selection of a few mRNA (**A**) transcripts, IGFBP-1 (**i**), GAPDH (**ii**), and HSPA8 (**iii**), of interest. RT-qPCR validation of RNA sequencing for miRNA (**B**) transcript miR-12136 (**i**) is also shown. Reverse transcription and amplification of cDNA was performed and measured using SYBR green fluorescence. Bar graphs show mean ± SD, controls arbitrarily assigned to 1 for comparison, *p*-values determined using unpaired *t*-test for RT-qPCR. *n* = 3 all, *p* < 0.05 considered significant, where * ≤ 0.05, *** ≤ 0.001, **** ≤ 0.0001.

Dysregulation of the PI3K-AKT-mTOR pathway may result in aberrant cell proliferation, metabolism and survival [7,50]. The precise relation of this signaling pathway to regulation by miRNAs is not fully understood. It is worth noting that there is a possibility of incomplete annotation for some genes and miRNAs, as we used a validated target database for our analysis, and many of the miRNAs hav poorly characterized functions. To validate our sequencing data, we chose some highly expressed and relevant miRNAs and mRNAs and validated the sequencing data by RT-qPCR. As expected, the changes were similar: IGFBP-1 mRNA levels were increased in hypoxia by 6.2-fold (*p* = 0.002) in RT-qPCR and 6.7-fold (*p* < 0.001) in RNA sequencing data (Figure 2A(i)). GAPDH also had similar fold changes in hypoxia: +3.2-fold (*p* < 0.001) in RNA seq, +2.0-fold (*p* < 0.001) in RT-qPCR (Figure 2A(ii)). We additionally compared fold changes in HSPA8 in hypoxia: −3.4-fold (*p* < 0.001) in RNAseq and −4.4-fold (*p* < 0.001) in RT-qPCR (Figure 2A(iii)). Similarly, miR-12136 levels were changed in hypoxia by −1.3-fold (*p* = 0.014) in RNAseq and −1.3-fold (*p* < 0.001) in RT-qPCR (Figure 2B(i)).

**Figure 3:**
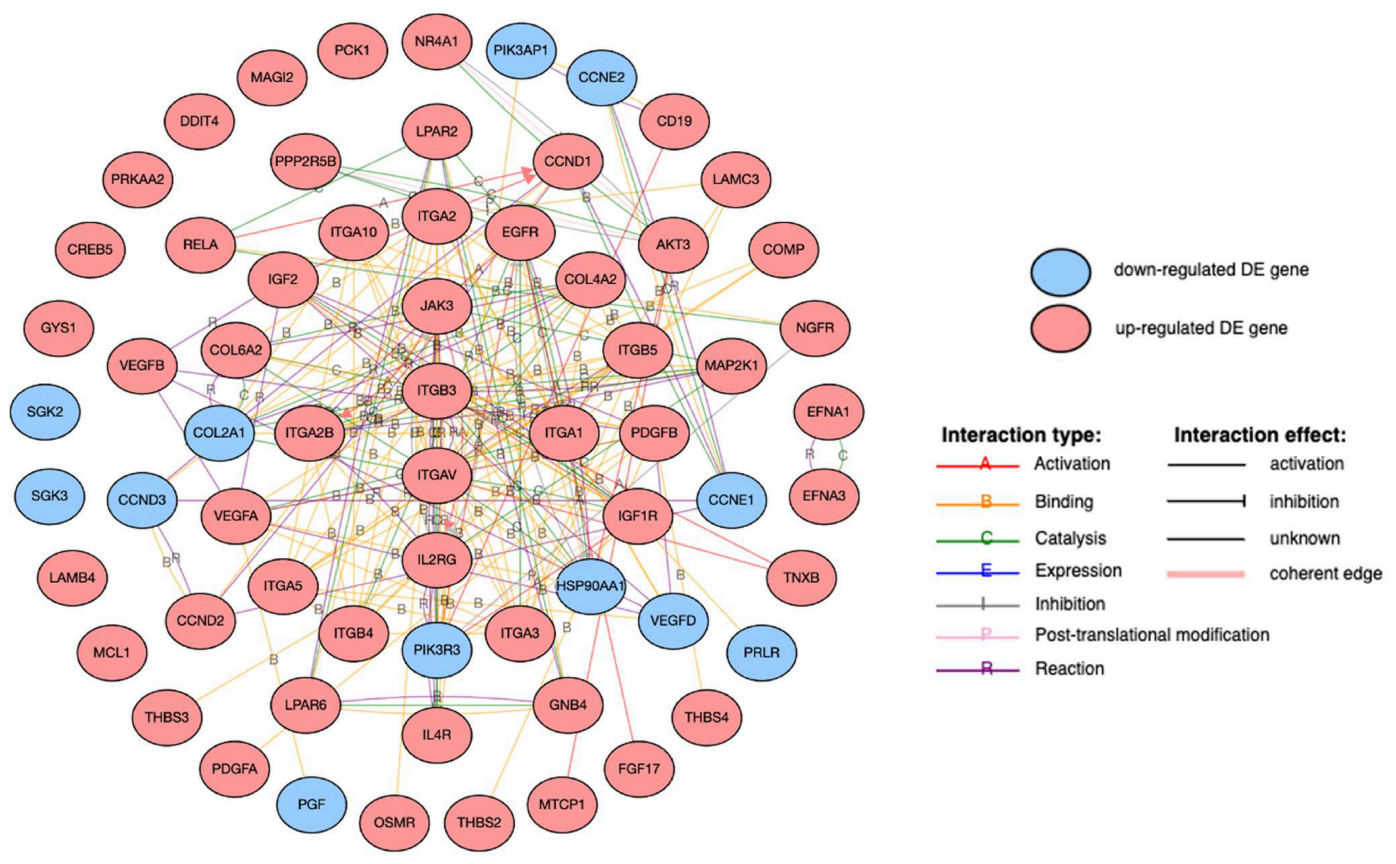
Network visualization of interconnected genes involved in PI3K-Akt signaling differentially expressed in response to hypoxia, using iPathwayGuide. The hub diagram shows a comprehensive interaction map of genes involved in the respective pathways. Nodes represent differentially expressed genes in the network and are color-coded to show upregulation (red) or downregulation (blue) and lines connecting nodes define the interaction.

We observed mostly upregulation (red) of genes associated with the PI3K-AKT signaling pathway in hypoxia (Figure 3), generated using iPathwayGuide ^TM^ (AdvaitaBio Corporation) [51,52]. Notably, the hub genes integrin subunit beta 3 (ITGB3) and integrin αv (ITGAV) demonstrated the highest connectivity in the network. ITGB3 is a crucial regulator within the tumor immune microenvironment, promoting the occurrence, development, and invasion of various carcinomas [53] and studies show a strong correlation between ITGB3 and tumor drug resistance [54]. These integrins play a crucial role in angiogenesis and vascular remodeling under hypoxia [55], which is supported by increased levels of vascular endothelial growth factor A (VEGFA, +11-fold, *p* < 0.001), which promotes vascularization. Other major genes involved in this pathway were also significantly altered in hypoxia; phosphoinositide-3-kinase regulatory subunit 3 (PIK3R3) was downregulated (−17-fold, *p* < 0.001), while AKT3 was upregulated (+3.8-fold, *p* < 0.001). These genes are involved in insulin signaling, and disturbance of this pathway can result in altered growth and insulin resistance [56].

### 2.3. The miRNA–mRNA Interaction Network Highlights Potential Modulators of mRNA Levels

**Figure 4:**
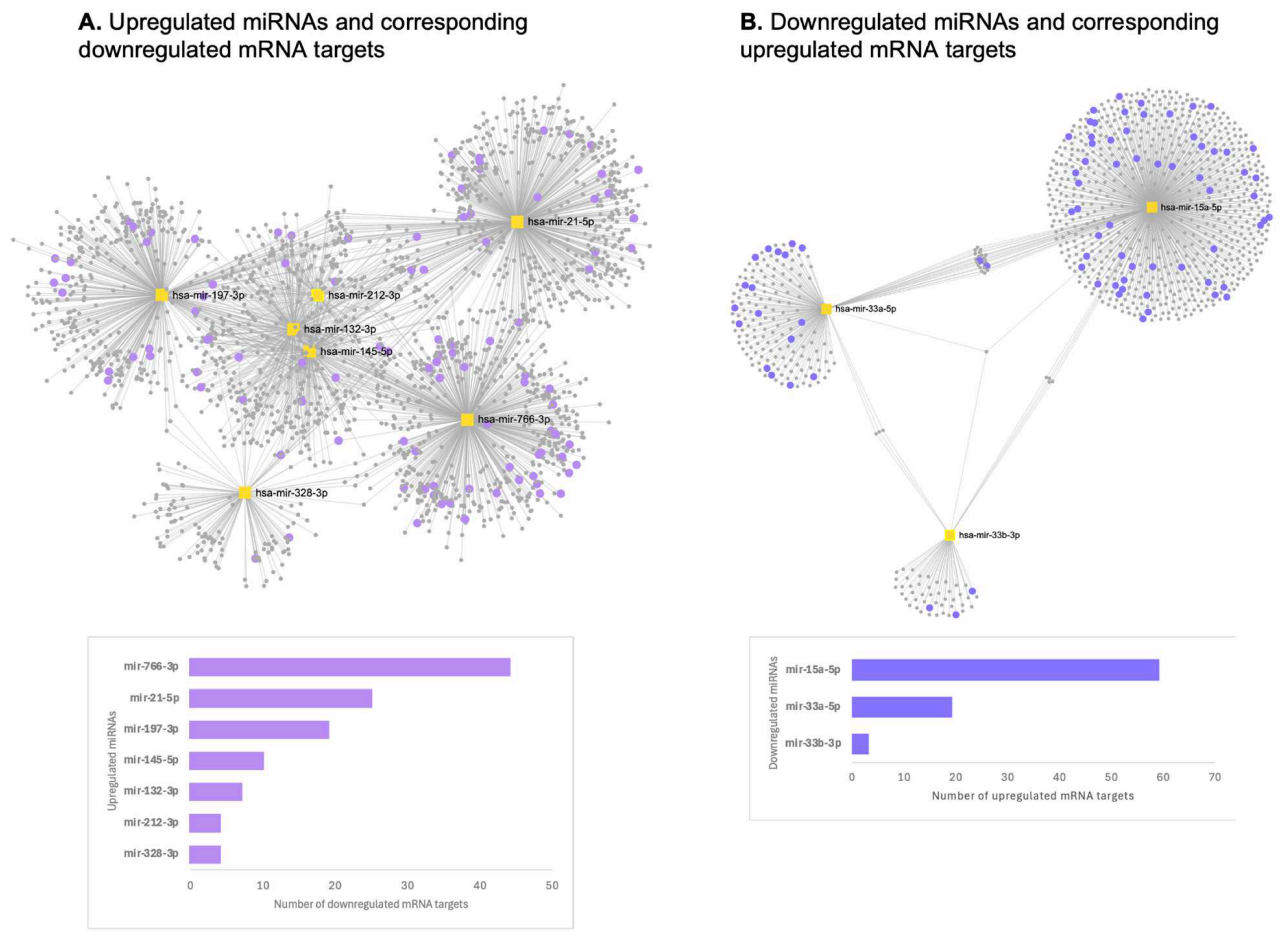
mRNA–miRNA interaction network of significantly differently expressed transcripts. Differentially expressed mRNA and miRNA in hypoxia are shown in an interaction network, with upregulated miRNAs and corresponding downregulated mRNA targets (**A**), downregulated miRNAs, and corresponding upregulated mRNA targets (**B**) in hypoxia compared to normoxia. The miRNA–mRNA network was created using miRNet 2.0, with selection filters for experimentally verified interactions in human liver; target genes were obtained from miRTarBase v8.0. Yellow squares denote miRNA nodes. Circles represent target genes, while purple specifies differentially expressed gene targets. Graphs are shown below each network with the DE miRNAs in hypoxia as well as the number of corresponding targets differentially regulated in hypoxia.

To identify mRNAs that are altered in abundance due to regulation by miRNA, we created a miRNA–mRNA interaction network using differentially expressed mRNAs and miRNAs. This network was created using miRNet [57], with interactions specific to *Homo sapiens* liver. Only interactions experimentally validated in the literature and present in the miRTarBase v8.0 [58] are shown. Looking at the interactions between upregulated miRNAs and downregulated corresponding mRNA targets (Figure 4A), many genes targeted by upregulated miRNAs in hypoxia are involved in lipid metabolism and steroid biosynthesis. Glycerol-3-phosphate acyltransferase (GPAM, −3.2-fold, *p* < 0.001) is likely a target of miR-197-3p (+1.9-fold, *p* < 0.001), while patatin-like phospholipase domain-containing 3 (PNPLA3, −2.9-fold, *p* < 0.001) is targeted by miR-766-3p (+2.1-fold, *p* < 0.001) (Table S8).

We additionally investigated several miRNAs which were decreased in abundance in hypoxia, and their respective increased mRNA targets (Figure 4B). Congruent with what was observed in Figure 1C, transcripts of genes involved in glycolysis and gluconeogenesis were increased, including lactate dehydrogenase A (LDHA, +2.3-fold, *p* < 0.001), while levels of an upstream miRNA that targets it, miR-33a-5p, was decreased (−1.6-fold, *p* < 0.001). miR-15a-5p was similarly decreased (−1.6-fold, *p* < 0.001), while its target, an aldehyde dehydrogenase (ALDH3B1) involved in glycolysis had increased expression (+2.7-fold, *p* < 0.001) (Table S9).

### 2.4. miRNAs Alter mTOR-IGFBP-1 Signaling

Four differentially regulated miRNAs were identified by RNAseq (miR-12136, miR-1248, miR-664a-5p, miR-6834-3p) with a potential role in mechanistic target of rapamycin (mTOR) signaling and insulin-like growth factor binding protein-1 (IGFBP-1) phosphorylation. The MicroRNA Target Prediction Database (miRDB, developed at University of Illinois, Chicago, USA) [59] was used to screen selected miRNAs and determine their predicted mRNA targets as shown in the literature. mTOR plays an important role in fetal growth and development; mTORC1 increases cell proliferation and protein transcription [7,60], while mTORC2 is involved in cell survival and metabolism. IGFBP-1 hyperphosphorylation in response to hypoxia in HepG2 cells is mediated by mTOR inhibition [31] and increased IGFBP-1 phosphorylation is linked to altered growth and metabolism.

miR-12136 was downregulated (−1.3-fold, *p* < 0.001) in hypoxia, associated with altering mRNA genes that regulate histone/RNA-binding. Although the role of this miRNA has been strongly associated with pre-eclampsia by altering the methylation at a specific site [61], whether it mediates other histone-modifying events is not known. miR-1248 is unchanged in hypoxia and is poorly studied in the context of mTOR signaling, but has been previously shown to target Raf1, which can impact PI3K-Akt signaling via TSC1/2 inhibition [7]. However, studies on miR-1248 in cancer progression tell an incomplete story, as elevated levels have been proposed as a biomarker associated with disease progression in pediatric low-grade gliomas [62], while other studies suggest miR-1248 has a protective effect by acting as a tumor suppressor gene in CRC [63]. Its role in hepatocellular carcinoma is not known. miR-664a was increased (+1.5-fold, *p* < 0.001) in hypoxia and may alter the activity of HIF-1 as a transcription factor. Interestingly, the insulin receptor substrate 1 (IRS1) gene is a target of miR-664a, which can activate PI3K, therefore affecting mTOR signaling. The transfection of miR-664 mimics suppressed cell proliferation in breast cancer cells [64]. miR-6834 was unchanged (−1.8-fold, *p* = 0.2) in hypoxia and is not well-studied; however, elevated expression of its target, thymidylate synthetase (TYMS), is associated with poor prognosis for nasopharyngeal carcinoma [65]. Interestingly, we see significantly decreased TYMS levels (−1.7-fold, *p* < 0.01) despite unchanged miR-6834 in hypoxia, suggesting alternate methods of regulation. Recent work shows TYMS directly impacts the AMP kinase (AMPK)-mTOR signaling pathway [66]. We, therefore, chose to explore the effects of the upregulation of these miRNAs on mTOR signaling and IGFBP-1 phosphorylation as an attempt to elucidate possible functions.

### 2.5. miRNA Transfection into HepG2 Cells Alters Kinase Transcript Levels

miRNAs can decrease translation of mRNAs by either translationally silencing mRNAs or promoting their degradation. To determine the impact of miRNAs on mRNA translation and protein levels, we transfected HepG2 cells with miRNAs of interest, a negative control with no miRNA (Ctr), and a negative control with scrambled RNA (Scr). To confirm miRNA transfection resulted in sustained overabundance of the transfected miRNAs, we measured levels at the time of collection, using RT-qPCR (Figure S2), and miRNAs were increased as follows: miR-12136 (+3.7-fold, *p* = 0.017), miR-1248 (+6000-fold, *p* = 0.002), miR-664a-5p (+8.6-fold, *p* = 0.025), and miR-6834-3p (+2E6-fold, *p* = 0.010).

**Figure 5:**
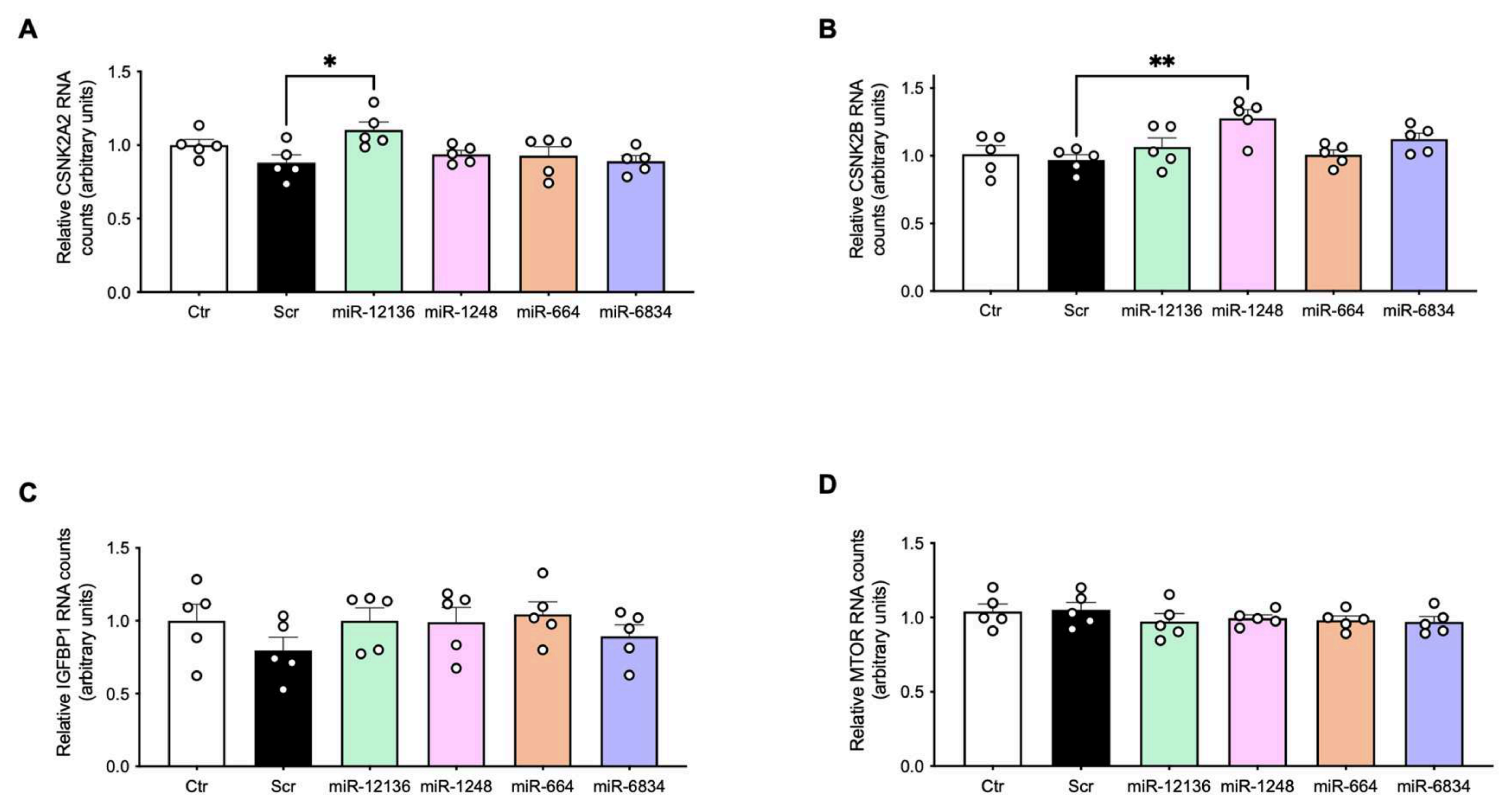
Transfection of miRNA into HepG2 cells alters kinase transcript levels. RT-qPCR quantified RNA counts of CNSK2A2 (**A**), CNSK2B (**B**), IGFBP1 (**C**) and MTOR (**D**) secretion in HepG2 cells transfected with miRNAs (40 nM, *n* = 5). Bar graphs show mean ± SD, controls arbitrarily assigned to 1 for comparison, analyzed with one-way ANOVA, *n* = 5 all, *p* < 0.05 is considered significant, where * ≤ 0.05, ** ≤ 0.01.

Casein kinase 2 (CK2) is composed of two catalytic subunits (α and α′) and two regulatory β subunits and is involved in cell growth and proliferation. Although the mechanisms of phosphorylation of IGFBP-1 at Ser101 and Ser119 are not known, previous studies have implicated CK2 in the process [67]. CK2 overexpression is associated with the poor prognosis of many cancers, due to its involvement in apoptosis inhibition, DNA damage response, and cell cycle regulation [68]. To characterize the relationship between CK2 and the highly abundant miRNAs, we measured CK2 subunit transcript abundance. We found that only the transfection of miR-12136 increased transcript levels of CSNK2A2 (+1.3-fold, *p* = 0.011, Figure 5A) and the gene encoding CK2 α’. Interestingly, miR-12136 overexpression had no effect on the transcript abundance for its beta subunit CK2β, CSNK2B. On the other hand, CSNK2B levels were increased (+1.3-fold, *p* = 0.002, Figure 5B) following transfection of miR-1248, whereas CSNK2A2 remained unchanged. Transfection of any of the four chosen miRNAs had no effect on either IGFBP1 (Figure 5C) or MTOR (Figure 5D) transcript levels.

### 2.6. miRNA Transfection into HepG2 Cells Alters mTOR-IGFBP-1 Signaling at the RNA and Protein Level

Using RNA sequencing, we observed that one of the most altered pathways was PI3K-Akt signaling, and IGFBP1 transcript abundance was increased (+6.8-fold, *p* < 0.001) in hypoxia. We have previously shown that hypoxia inhibits mTORC1 activity, which is linked to poor fetal development [69]. In this study, we use phosphorylation of 4E-BP1 at Thr70 as a functional readout for mTORC1 activity. Using Western blotting, we investigated whether IGFBP-1 phosphorylation at the two sites (Ser101 and Ser119) linked to decreased growth [21,31] was altered by testing secreted IGFBP-1 from cell media.

**Figure 6:**
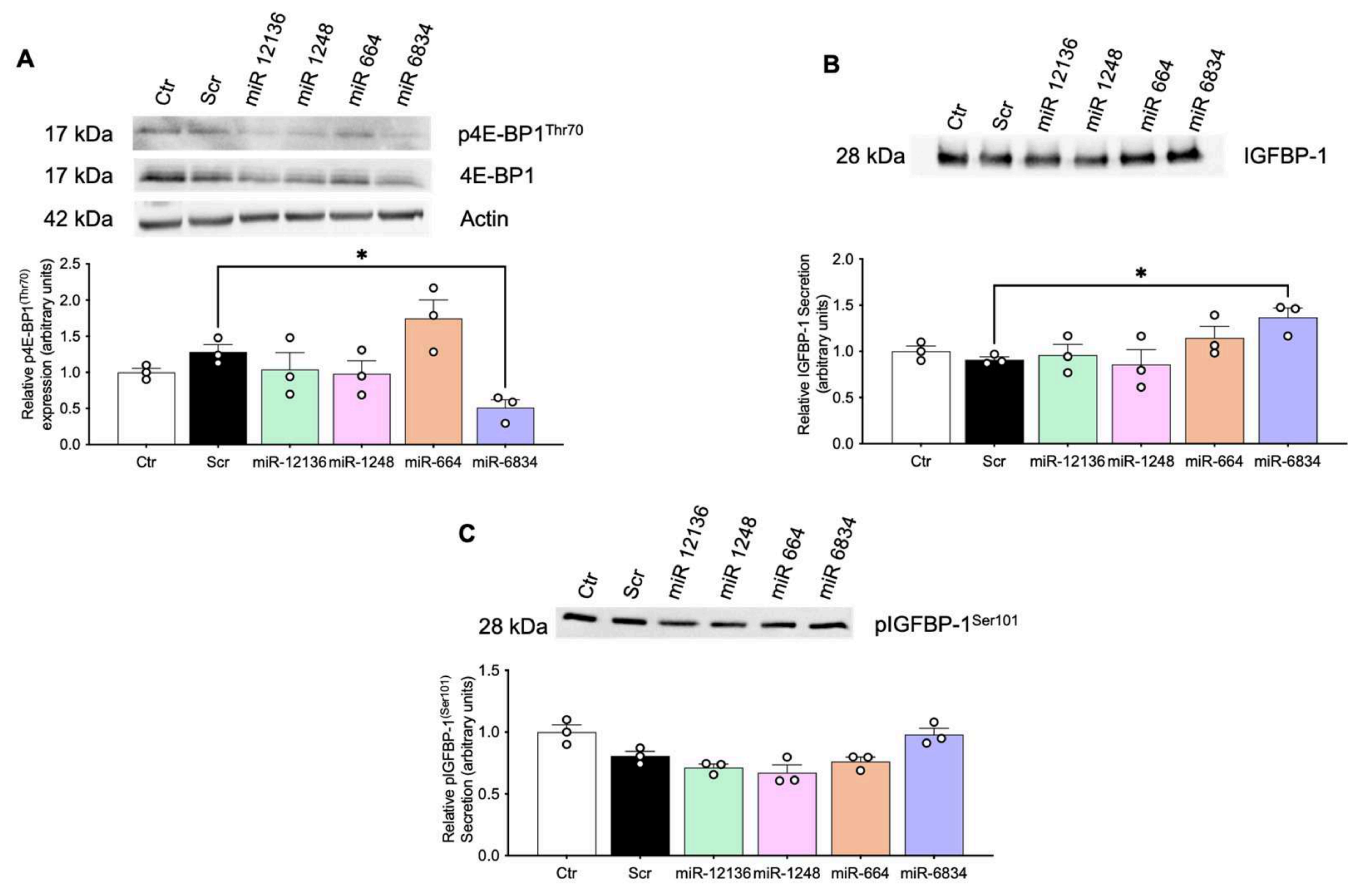
Transfection of miRNA into HepG2 cells alters mTORC1 activity and IGFBP-1 secretion and phosphorylation. Representative Western blot of mTORC1 functional readout p4EBP1^Thr70^ (**A**) expression in lysate and secretion of IGFBP-1 (**B**) and pIGFBP-1^Ser101^ (**C**) in media collected from HepG2 cells transfected with miRNAs (40 nM, *n* = 3 all) under standard culture conditions. Western blots were quantified with bar graphs showing mean ± SD, controls arbitrarily assigned to 1 for comparison, analyzed with one-way ANOVA, *p* < 0.05 is considered significant, where * ≤ 0.05. Equal loading of protein (50 µg) or media (20–40 µL) was performed.

Transfection with three of the four tested miRNAs, miR-12136, miR-664 and miR-1248 did not result in changes to mTORC1 activity as measured by phosphorylation of its substrate 4E-BP1 at Thr70. No change in secreted IGFBP-1 protein abundance or phosphorylation was observed. In contrast, transfection of miR-6834 (Figure 6A) into HepG2 cells significantly inhibited mTORC1 activity, as seen by decreased 4E-BP1 phosphorylation (−0.60-fold, *p* = 0.031), and additionally increased IGFPB-1 secretion (+1.5-fold, *p* = 0.041, Figure 6B). Literature suggests involvement of miR-6834 in regulating TYMS, which is linked to altered mTOR signaling [65,66] in tumor progression. However, there was no change in IGFBP-1 phosphorylation at Ser101 in miR-6834 transfected cells (Figure 6C). These findings provide insight into the role of miRNAs in regulating mTOR-IGFBP-1 signaling but further analyses are needed to clarify the mechanisms underlying these effects.

## 3. Discussion

Hypoxia significantly impacted 20.2% of the transcriptome resulting in many altered cellular processes, including a shift to anaerobic glycolysis for energy production, reduced DNA replication and cell proliferation, and altered PI3K-Akt-mTOR signaling. Overall, these findings are consistent with hepatocellular carcinoma (HCC) research showing that hypoxia increases glucose uptake and anaerobic metabolism to compensate for decreased energy availability [70] through post-transcriptional mechanisms [71,72]. Additionally, hypoxia alters DNA replication and cellular metabolism, supported by previous studies showing hypoxia inhibits mTOR signaling [7,60] leading to changes in transcription and proliferation.

We have previously shown that hypoxia, through the activation of HIF-1 and regulated in development and DNA responses 1 (REDD1), inhibits mTORC1 activity, resulting in increased IGFBP-1 secretion and phosphorylation [69,73]. Under normoxic conditions, HIF-1α is rapidly degraded, but HIF-1α is stabilized in hypoxia, leading to transcriptional activation of multiple adaptive genes [50]. HIF-1 can upregulate genes involved in glucose metabolism, including glucose transporters and glycolytic enzymes, to ensure adequate ATP production in the absence of oxygen [74]. Under hypoxic conditions, cells increase their reliance on glycolysis to maintain ATP production despite suppressed oxidative phosphorylation. Our data reveals substantial changes in glycolysis-related gene expression in response to hypoxia, including HK2 and LDHA, consistent with previous findings, alongside the concurrent decreased level of miRNAs regulating these mRNA targets, including miR-199a-5p. In hypoxic HCC cells, HIF-1 regulation by miR-130a-3p mediates glycolysis, glucose consumption, lactate production and HK2 abundance [75]. The RNA-binding protein HuR prevents miR-199a maturation, which normally targets HK2, thus driving the glycolytic switch in hypoxia [76]. Enhanced glycolytic flux and upregulation of HIF-1 additionally promotes angiogenesis and vascularization to increase oxygen perfusion in hypoxic tissues [43,77], and we see increased levels of VEGFA, a transcriptional target of HIF-1 [78,79].

Oxidative stress can additionally impair cellular proliferation. We showed that hypoxia downregulates the expression of several genes involved in DNA replication and fork progression, including MCM2, POLE2/3, POLA2, and other polymerase genes. Hypoxic conditions result in replication stress, whereby chronic hypoxia can inhibit both initiation and elongation steps of DNA replication, and induce disassembly of the replisome, preventing efficient restart following reoxygenation [80]. The reduction in folate biosynthesis transcripts additionally constrains nucleotide supply and S-phase entry [49,81]. REDD1, induced by both hypoxia and DNA damage, is a stress-responsive protein that inhibits mTORC1 signaling under conditions of nutrient or energy deprivation [60], slowing down cellular proliferation. Overall, the activation of HIF-1 and REDD1 in response to hypoxia leads to a complex cellular response aimed at promoting oxygen delivery and maintaining cellular energy homeostasis.

Although not much is known about the role of miR-6834, in this study, we showed for the first time, that overexpression of miR-6834 in liver cells inhibits mTORC1 activity and increases IGFBP-1 secretion. Additionally, the transfection of miR-1248 and miR-12136 both increased mRNA levels of CSNK2A2 and CSNK2B transcripts, which encode CK2 subunits. CK2 is a prolific kinase with hundreds of substrates and a central kinase hub in cancer. CK2 is involved in translation, stress signaling, and oncogenic signaling. CSNK2A2 has been reported to promote hepatocellular carcinoma progression through NF-κB activation [82]. The mechanistic link between these miRNAs and CK2 subunit levels is not known and warrants further studies. One target of miR-1248 is CREB3, which is also phosphorylated by CK2 (Figure S3) to regulate the unfolded protein response, a stress response activated in hypoxia [83,84]. CK2 activity is not only related to cell growth but also neurological diseases, autoimmune disorders, and diabetes [85], and thus we cannot exclude the role of CK2 in prolonged hypoxic remodeling and nutrient deficiency.

This work presents a hypoxia-responsive miRNA–mRNA network that couples HIF-1-mediated metabolic reprogramming, replication stress, and mTOR-IGFBP-1 regulation in HepG2 cells. These findings are directly relevant to hepatocellular carcinoma biology and contribute to our knowledge of oxygen-deprivation responses described in poor fetal development, where reduced IGF and growth signaling, alongside altered translational control, are centrally present. These findings contribute to our understanding of the mRNA–miRNA networks and further studies may support the use of certain miRNAs as biomarkers or therapeutic targets in hypoxic pathophysiology.

## 4. Materials and Methods

### 4.1. Cell Culture and Treatment of HepG2 Cells

HepG2 cells (ATCC-HB-8065, Manassas, VA, USA) were cultured in DMEM-F-12 with 10% fetal bovine serum at 37 °C, 20% O_2_, 5% CO_2_. Following plating, cells were exposed to either standard culture conditions (20% O_2_, normoxic control) or hypoxia (1% O_2_) for 24 h, as previously established, to induce a robust hypoxic response [69]. We confirmed hypoxic conditions by upregulation of hypoxia markers, including the activation of HIF-1 (Figure S4). Full culturing and treatment details can be found in Supplementary Materials.

### 4.2. Cell Lysate Collection for Protein and RNA

Media was collected, and adherent cells were washed with DPBS (Cat #14190144, Thermo Fisher Scientific, Waltham, MA, USA) prior to lysis in buffer containing protease and phosphatase inhibitors (Cat #9803, Cell Signaling Technology, Danvers, MA, USA). Following sonication and centrifugation, cell lysates were stored at −20 °C. Alternatively, following treatment, RNA was extracted using a Qiagen miRNeasy kit (Cat #217004, Qiagen, Venlo, The Netherlands) as per manufacturer instructions.

### 4.3. Next-Generation Sequencing

RNA sequencing of mRNA and miRNA levels was performed at the London Regional Genomics Center (Robarts Research Institute, London, ON, Canada). Bulk RNA sequencing was performed on three biological replicates per condition (normoxia, *n* = 3; hypoxia, *n* = 3). Sequencing yielded approximately 28.6–31.8 million reads per sample. Files were analyzed using Partek Flow (Illumina, San Diego, CA, USA) [86]; complete sequencing details can be found in the Supplementary Materials. Read count and alignment data can be found in Table S10.

### 4.4. miRNA Synthesis and Transfection into HepG2 Cells

Custom miRNAs (sequences in Table S1) or control scrambled RNA was transfected into HepG2 cells exposed to standard culture conditions for 48 h. Cell media, cell lysate, and RNA were collected as per manufacturer instructions as stated above.

### 4.5. RT-qPCR

RT-qPCR reactions were performed as described previously [87]. Briefly, following extraction and quantification, 2 µg of total RNA was reverse transcribed. Primers are listed in Table S2. cDNA was amplified with PowerUp (Cat #A25742, Thermo Fisher Scientific) or PowerTrack SYBR Green (Cat #A46110, Thermo Fisher Scientific). RT-qPCR reactions were performed using the QuantStudio 3 Real-Time PCR System (Applied Biosystems, Thermo Fisher Scientific). Each sample was analyzed in technical duplicate with three independent biological replicates per experimental group. Ct values were normalized to the reference gene (GAPDH for mRNA and SNORD47 for miRNA analyses) using the ΔCt method, and relative gene expression was calculated using the ΔΔCt method. Relative expression values were normalized to the control group (arbitrarily assigned 1). Amplification and melt curves were examined to confirm specific amplification and the absence of non-specific products or primer-dimers.

### 4.6. Western Blotting

Cell lysate was quantified and equal amounts of total protein were used to determine phosphorylation and total expression of 4E-BP1 at Thr70 obtained from Cell Signaling Technology. β-actin was used as a loading control. Total IGFBP-1 and IGFBP-1 phosphorylation at Ser101 was quantified in cell media by loading equal volume. Blots were visualized with commercially available enhanced chemiluminescence reagents. Full details are in the Supplementary Materials.

### 4.7. Statistical Analyses

Densitometric analyses were performed using Image Lab software (v6.0, Bio-Rad, Hercules, CA, USA) and data was visualized using GraphPad Prism 10 (Boston, MA, USA). Treatment groups were normalized to controls and analyzed with unpaired *t*-test and one-way ANOVA; results were expressed as mean ± SD, with *p* < 0.05 considered significant.

## Data Availability

All data generated or analyzed during this study are included in this published article and its Supplementary Materials. Further inquiries can be directed to the corresponding author.

## Supplementary Materials

The following supporting information can be downloaded at https://www.mdpi.com/article/10.3390/ijms27156886/s1.
