# Supplementary material for: Adaptation of the Transcriptome and miRNAome in Response to Hepatocellular Hypoxia
Source: Int J Mol Sci. 2026 Aug 1;27(15):6886. doi: 10.3390/ijms27156886 (PMC13467575; doi:10.3390/ijms27156886)
Supplement: Supplementary file 1 [file ijms-27-06886-s001.zip › 4. Supplemental_methods.pdf]

Supplemental Materials and Methods

The following supplementary methods provide extended details on the experimental procedures used in this study.

Cell culture and treatment of HepG2 cells

HepG2 cells (ATCC-HB-8065) were cultured in Dulbecco's Modified Eagle Medium (DMEM)-F-12 (Cat # 11320033, Thermo Fisher Scientific, MA, USA), supplemented with 10% fetal bovine serum (Cat #10082147, FBS, Thermo Fisher Scientific). Cells were grown in atmospheric air (20% O_2 with 5% CO_2) at 37°C. Cells were then plated in 6-well plates at 400K cells/well and allowed to attach for 16-24 hours. Cells were subsequently placed either in room air as a control (labelled normoxia, in reference to normal HepG2 growth conditions) or placed within a hypoxic chamber flushed for 10 minutes with 1% O_2 gas for a hypoxia treatment for 24 hours. Although 20% O_2 and 1% O_2 are not physiologically accurate to fetal growth conditions in pregnancy, these levels of oxygen have been utilized majorly as normoxic and hypoxic growing conditions for HepG2 cells; indeed, exposure to 1% oxygen induces hypoxic signaling in HepG2 cells, whereas the activation of HIF-1 is absent at 20% O_2 [13,79].

Cell lysate collection for protein and RNA. Following treatment, cells were placed on ice and cell media was collected. Cells were then washed with Dulbecco's phosphate-buffered saline (Cat #D8537, DPBS, Sigma-Aldrich), and 150 μL lysis buffer (1:1,000 protease inhibitor cocktail (Cat #P8340, Sigma-Aldrich), 1:1,000 phosphatase inhibitor cocktail 2 (Cat #P5726, Sigma-Aldrich), 1:1,000 phosphatase inhibitor cocktail 3 (Cat #P0044, Sigma-Aldrich)), with 10x cell lysis buffer (Cat #9803, Cell Signaling Technologies) in water were additionally added to each well, for use in western blotting. Cells were frozen at -80°C overnight, and subsequently scraped, sonicated for 45 seconds, and centrifuged for 30 mins at 10,000 x g. The cell lysates were stored at -20°C until analysis. Alternatively, miRNA and mRNA were extracted with the Qiagen miRNeasy kit (Cat #217604, Qiagen, Hilden, Germany) as per manufacturer instructions. Extracted RNA was stored at -80°C until analysis.

Next Generation sequencing. Total RNA samples from HepG2 cells treated with or without hypoxia were quantified using the NanoDrop (Thermo Fisher Scientific, Waltham, MA) and quality was assessed using 1 μL (50-500 ng/μL) of sample on the Agilent 2100 Bioanalyzer (Agilent Technologies Inc., Palo Alto, CA) and the RNA 6000 Nano kit (Caliper Life Sciences, Mountain View, CA). RNA sequencing of mRNA and miRNA levels was performed at the London Regional Genomics Centre (Robarts Research Institute, London, Ontario, Canada) using the Illumina NextSeq 500 (Illumina Inc., San Diego, CA).

miRNA sequencing and data analysis. miRNA-seq libraries were made using the QIAseq miRNA Library Kit (Qiagen, Hilden, Germany). Briefly, samples underwent ligation, cDNA synthesis, amplification with indexed primers, and clean-up. Libraries were then equimolar pooled into one library and size distribution was assessed on an Agilent High Sensitivity DNA Bioanalyzer chip and quantified using the Qubit 2.0 Fluorimeter (Thermo Fisher Scientific, Waltham, MA). The library was sequenced on an Illumina NextSeq 500 as 76 bp single end run, using one Mid-Output v2 kit (150 cycles). Fastq data files were analyzed using Partek Flow (St. Louis, MO). After importation, reads were trimmed to 18 bp in length, data was aligned to the Homo sapiens hg38 genome using Bowtie 1.0.0 and annotated using miRBase mature microRNAs version 22 deduplicated. Features with less than 7 reads were filtered out, followed by normalization by cpm (counts per million and add 1.0). Fold-change and p-values were determined using Partek Flow's Gene Specific Analysis (GSA). Filtered lists of miRNAs changing greater than 1.5-fold and with a p-value of less than 0.05 between group D vs W were then analyzed for enriched Gene Ontology (GO) terms and KEGG pathways, based on TargetScan 7.2 annotations.

mRNA sequencing and data analysis. Total RNA samples were processed using the Vazyme VAHTS Total RNA-seq (H/M/R) Library Prep Kit for Illumina (Vazyme, Nanjing, China), which includes rRNA reduction. Briefly, samples were rRNA depleted, fragmented, cDNA was synthesized, indexed, cleaned up and amplified via PCR. Libraries were then equimolar pooled into one library and size distribution was assessed on an Agilent High Sensitivity DNA Bioanalyzer chip and quantified using the Qubit 2.0 Fluorimeter (Thermo Fisher Scientific, Waltham, MA). The library was sequenced on an Illumina NextSeq 500 as a 76 bp single end run, using one High Output v2 kit (75 cycles). Fastq data files were analyzed using Partek Flow (St. Louis, MO). After importation, data was aligned to the *Homo sapiens* hg38 genome using STAR 2.7.3a and annotated using Ensembl Transcripts release 102. Features with more than 12 reads were normalized using cpm (counts per million and add 1.0). Fold change and p-values were determined using Partek Flow's Gene Specific Analysis (GSA). Filtered gene lists were then analyzed for enriched Gene Ontology (GO) and KEGG pathway terms.

miRNA and mRNA integration. Filtered miRNA and mRNA lists were imported into Partek Genomics Suite v7.2 (St. Louis, MO) and combined and correlated using TargetScan 7.2 annotations. Functional analysis was perform using iPathwayGuide platform (Advaita Bioinformatics, Ann Arbor, MI).

miRNA synthesis and transfection into HepG2 cells. miRNAs were ordered from Sigma-Aldrich (St. Louis, MO) as custom RNA oligos; sequences are found in **Supplemental Table S1**. RNA oligos were ordered dry and desalted with a 5′ phosphate. Synthesized miRNAs (30 nM) or control scrambled RNA (30 nM, Cat #SIC001, Sigma-Aldrich) were transfected into HepG2 cells

using Dharmafect transfection reagent 4 (Cat #T-2004, Thermo Fisher Scientific) for 48 hours, in 10% FBS DMEM F-12 media. A negative control containing no transfected RNA was also used for comparisons. Both cell media and cell lysate were collected for analyses, and the efficiency of target silencing was determined at the protein level using western blot analysis. miRNA and mRNA were extracted with the Qiagen miRNeasy kit (Cat #217604) as per manufacturer instructions. Extracted RNA was stored at -80°C until analysis.

Reverse transcription – quantitative polymerase chain reaction (RT-qPCR). RT-qPCR reactions were performed as described previously [33]. Briefly, following extraction by the miRNeasy kit (Qiagen) and quantification by a Nanodrop 2000 spectrophotometer (Thermo Fisher Scientific), 2 μg of total RNA was reverse transcribed using High-Capacity cDNA Reverse Transcription Kit (Applied Biosystems, Waltham, MA, USA) for each reaction. Primers are listed in **Supplemental Table S2**. The cDNA was amplified with PowerUp or PowerTrack SYBR Green (Applied Biosystems) on QuantStudio 3 Real-Time PCR System, with 3 biological replicates and 2 technical replicates performed per sample. For each target, the control without reverse transcriptase (no RT control) was used to exclude false-positive amplification.

SDS PAGE & western blotting. Cell lysate was quantified and equal amounts of total protein (50 - 100 μg) were used to determine phosphorylation and total expression of 4E-BP1 (Cat# 9644) at Thr70 (Cat# 9455) obtained from Cell Signaling Technology. To confirm equal loading, β-actin (Cat# 3700, Cell Signaling Technology) was used as a loading control during quantification. An IGFBP-1 polyclonal antibody was a gift from Dr. Robert Baxter (Kolling Institute of Medical Research, Sydney, Australia). Custom IGFBP-1 polyclonal antibody targeting Ser101 was generated at YenZym Antibodies LLC (CA, USA). Equal volume loading of cell media was performed to determine IGFBP-1 secretion and phosphorylation at Ser101. Blots were blocked with 5% skim milk in Tris-buffered saline plus 0.1% Tween-20 (TBS-T; Cat #TWN510, BioShop) for 1 hour at room temperature. Primary antibodies were used at a dilution of 1:1,000 except for total IGFBP-1 antibody at 1:10,000, pIGFBP-1^{Ser101} at 1:500, and β-actin at 1:3,000. Cells were probed with primary antibodies overnight at 4°C. Peroxidase-labeled anti-mouse (Cat# 1706516, Bio-Rad Laboratories, CA, USA) or anti-rabbit (Cat# 1706515, Bio-Rad Laboratories) antibodies (1:10,000) were used as secondary antibodies. Secondary antibody was applied for 1 hour at room temperature and blots were subsequently visualized by Clarity enhanced chemiluminescence (ECL) reagents (Cat #1705060, Bio-Rad) and imaged on a ChemiDoc MP Imager (Bio-Rad).

Statistical analyses. Western blot band intensities were quantified with densitometry using Image Lab software (Bio-Rad), and statistical analyses were performed using GraphPad Prism 10 (Graph Pad Software, MA, USA). Treatment groups were normalized to controls and controls were assigned an arbitrary value of 1 to facilitate comparisons. Quantification of RNAs was performed by ΔΔCT method, using SNORD47 as reference for miRNAs and actin for mRNAs.

Unpaired t-test and one-way ANOVA were used to compare means and determine significance; results were expressed as mean ± SD, with $p<0.05$ considered significant.
