# Supplementary material for: Adaptation of the Transcriptome and miRNAome in Response to Hepatocellular Hypoxia
Source: Int J Mol Sci. 2026 Aug 1;27(15):6886. doi: 10.3390/ijms27156886 (PMC13467575; doi:10.3390/ijms27156886)
Supplement: Supplementary file 1 [file ijms-27-06886-s001.zip › 6. Kakadia miRNA 2025 Supplemental Figures JK.pdf]

Supplemental Figures

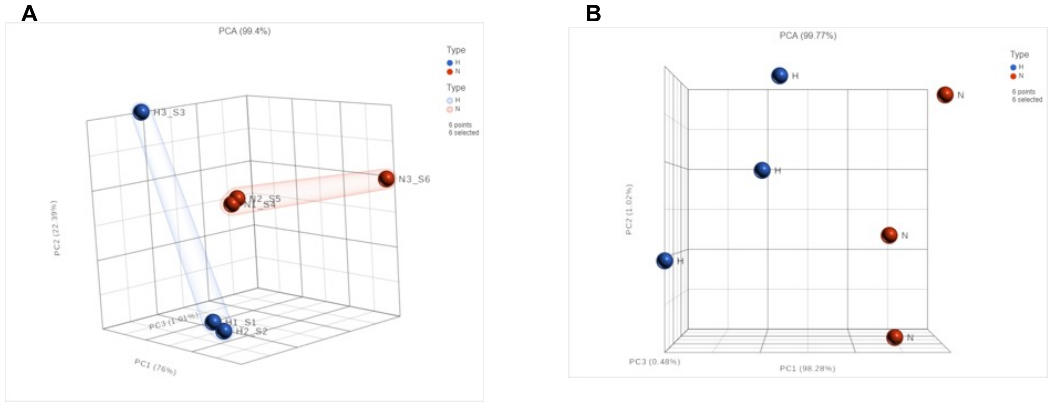

Supplemental Figure S1. Principal component analysis of samples used for mRNA (A) and miRNA (B) analyses.

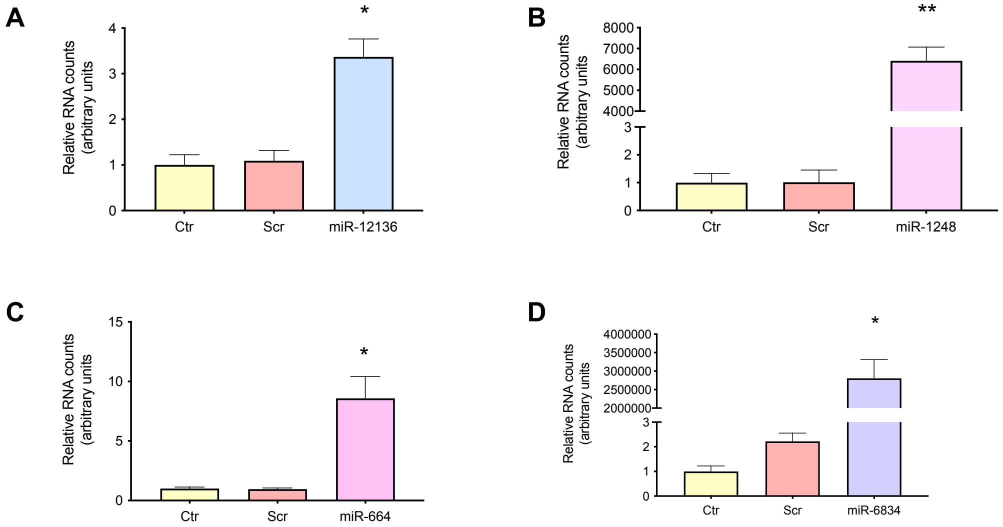

Supplemental Figure S2. Validation of miRNA transfection efficacy.
RT-qPCR quantified RNA counts of mir-12136 (**A**), mir-1248 (**B**), mir-664a (**C**) and mir-6834 (**D**) levels in HepG2 cells transfected with respective miRNAs (40 nM, n=3). Bar graphs show mean ± SD, controls arbitrarily assigned to 1 for comparison, analyzed with one-way ANOVA, $p < 0.05$ is considered significant.

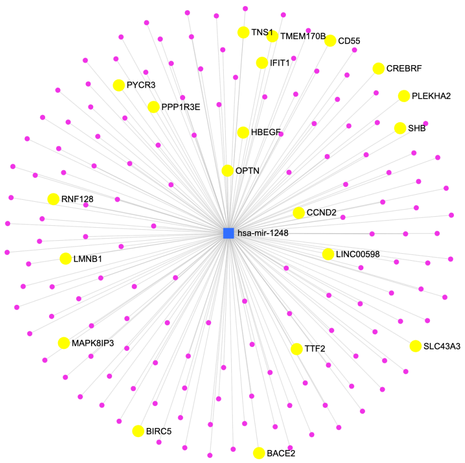

Supplemental Figure S3. Differentially expressed targets of miR-1248 in hypoxia. Blue node at the center represents miRNA of interest mir-1248. Known targets shown as colored circles, validated by miRTarBase v8.0. Yellow highlights denote differentially expressed gene targets.

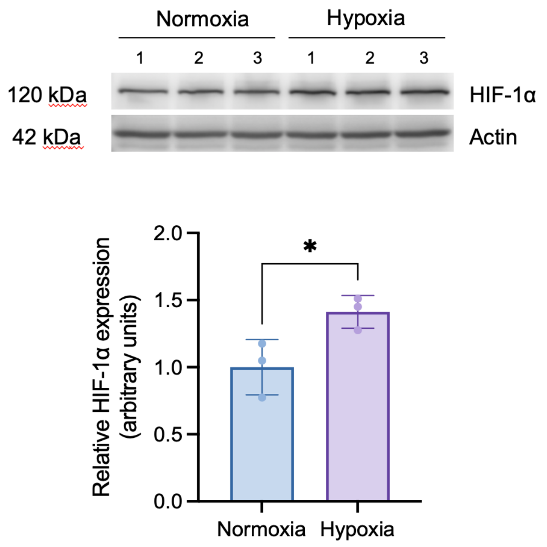

Supplemental Figure S4. Validation of HIF-1 activation in hypoxia.

Western blot of HIF-1α expression in cellular lysate collected from HepG2 cells in either standard culture conditions (normoxia) or 1% O_2 after 24 hours (n=3). Bar graphs show mean $\underline{+}$ SD, controls arbitrarily assigned to 1 for comparison, analyzed with unpaired *t*-test, $p < 0.05$ is considered significant.

Supplemental Table S1. miRNA oligos used for transfection into HepG2 cells.

Primer name	Sequence
**hsa-miR-1248**	5'-ACCUUCUUGUAUAAGCACUGUGCUAAA-3'
**hsa-miR-664a-5p**	5'-ACUGGCUAGGGAAAAUGAUUGGAU-3'
**hsa-miR-6834**	5'-GUGAGGGACUGGGAUUUGUGG-3'
**hsa-miR-12136**	5'-GAAAAAGUCAUGGAGGCC-3'

Supplemental Table S2. mRNA and miRNA primers.

Primer name	Step	Sequence
**Universal reverse miRNA**	PCR	5′-CCA GTG CAG GGT CCG AGG TA-3′
**hsa-miR-12136**	RT	5’-GTC GTA TCC AGT GCA GGG TCC GAG GTA TTC GCA CTG GAT ACG AC GGC CTC-3’
**hsa-miR-12136 fwd**	PCR	5’-AGGCGCGAAAAAGTCATGGAG-3’
**hsa-miR-1248**	RT	5’-GTC GTA TCC AGT GCA GGG TCC GAG GTA TTC GCA CTG GAT ACG AC TTT AGC-3’
**hsa-miR-1248 fwd**	PCR	5’-ACGCGCACCTTCTTGTATAAG-3’
**hsa-miR-664a-5p**	RT	5’-GTC GTA TCC AGT GCA GGG TCC GAG GTA TTC GCA CTG GAT ACG AC ATC CAA-3’
**hsa-miR-664a-5p fwd**	PCR	5’-AGGCAGACTGGCTAGGGAAAA-3’
**hsa-miR-6834-3p**	RT	5’-GTC GTA TCC AGT GCA GGG TCC GAG GTA TTC GCA CTG GAT ACG AC CCA CAA-3’
**hsa-miR-6834-3p fwd**	PCR	5’-AGGCGTGAGGGACTGGGAT-3’
**hsa-miR-381**	RT	5’-GTC GTA TCC AGT GCA GGG TCC GAG GTA TTC GCA CTG GAT ACG AC ACA GAG-3’
**hsa-miR-381 fwd**	PCR	5’-ATTCGCTATACAAGGGCAAGC-3’
**hsa-miR-365a-3p**	RT	5’-GTC GTA TCC AGT GCA GGG TCC GAG GTA TTC GCA CTG GAT ACG AC ATA AGG-3’
**hsa-miR-365a-3p**	PCR	5’-CGGCGATAATGCCCCTAAAAA-3’
**hsa-miR-203a**	RT	5’-GTC GTA TCC AGT GCA GGG TCC GAG GTA TTC GCA CTG GAT ACG AC CTA GTG-3’
**hsa-miR-203a fwd**	PCR	5’-CAGGCGGTGAAATGTTTAGGA-3’
**Beta actin**	PCR	F: 5’-CACCAACTGGGACGACAT-3’<br>R: 5’-ACAGCCTGGATAGCAACG-3’
**CSNK2A2**	PCR	F: 5’-CCCGAGCTGGGGTAATCAAG-3’<br>R: 5’-CCACCACGAAGGTTCTCCAG-3’
**CSNK2B**	PCR	F: 5’-GAGCAGGTCCCTCACTATCGAC-3’<br>R: 5’-CTTCACCTGGGATGTCAATGGG-3’
**IGFBP1**	PCR	F: 5’-CATCCTTTGGGACGCCATC-3’<br>R: 5’-GGATGTCTCACACTGTCTGC-3’
**MTOR**	PCR	F: 5’-AGCATCGGATGCTTAGGAGTGG-3’<br>R: 5’-CAGCCAGTCATCTTTGGAGACC-3’
**GAPDH**	PCR	F: 5’-GAAGGTGAAGGTCGGAGTCAAC-3’<br>R: 5’-CAGAGTTAAAAGCAGCCCTGGT-3’
**HSPA8**	PCR	F: 5’-GCTGCTGCTATTGCTTACGG-3’<br>R: 5’-GCTGGAAGAGAGGGTACGC-3’

Supplemental Table S3. Overview of differentially expressed mRNAs and miRNAs

	Total # detected	Fold change cut-off	# Significantly increased (H vs N)	# Significantly decreased (H vs N)
**mRNA**	20241	2-fold	2667	1423
**miRNA**	1365	1.5-fold	80	64

Supplemental Table S4. mRNAs significantly decreased in abundance (>2.0-fold) in hypoxia.

Supplemental Table S5. mRNAs significantly increased in abundance (>2.0-fold) in hypoxia.

Supplemental Table S6. miRNAs significantly decreased in abundance (>1.5-fold) in hypoxia.

microRNA ID	p-value	FDR	Fold change (H vs. N)	microRNA ID	p-value	FDR	Fold change (H vs N)
**hsa-miR-4455**	6.82E-20	2.42E-18	-9.82	**hsa-miR-1236-3p**	3.40E-02	1.15E-01	-1.87
**hsa-miR-5088-3p**	6.09E-04	3.46E-03	-9.12	**hsa-miR-6765-3p**	3.06E-22	1.24E-20	-1.86
**hsa-miR-4709-3p**	8.81E-34	5.59E-32	-7.81	**hsa-miR-4677-5p**	2.35E-02	8.54E-02	-1.85
**hsa-miR-4766-5p**	1.38E-19	4.70E-18	-6.55	**hsa-miR-3182**	1.21E-14	2.83E-13	-1.74
**hsa-miR-12135**	7.84E-188	2.32E-185	-5.82	**hsa-miR-15b-5p**	3.58E-29	1.87E-27	-1.73
**hsa-miR-12130**	1.03E-114	1.83E-112	-5.78	**hsa-miR-3123**	1.52E-06	1.38E-05	-1.73
**hsa-miR-381-5p**	1.41E-08	1.69E-07	-5.20	**hsa-miR-33b-3p**	3.44E-02	1.15E-01	-1.73
**hsa-miR-7108-5p**	6.65E-03	2.97E-02	-4.48	**hsa-miR-25-5p**	5.88E-07	5.68E-06	-1.68
**hsa-miR-3186-5p**	2.09E-03	1.09E-02	-4.38	**hsa-miR-545-5p**	5.36E-05	3.75E-04	-1.68
**hsa-miR-4454**	6.30E-88	6.99E-86	-3.96	**hsa-miR-219a-1-3p**	1.51E-02	5.97E-02	-1.68
**hsa-miR-6867-5p**	1.52E-04	1.00E-03	-3.62	**hsa-miR-1294**	1.62E-02	6.31E-02	-1.66
**hsa-miR-4483**	1.90E-134	4.23E-132	-3.57	**hsa-miR-3611**	7.44E-03	3.22E-02	-1.65
**hsa-miR-4458**	2.87E-26	1.27E-24	-3.55	**hsa-miR-33a-5p**	2.55E-08	3.01E-07	-1.62
**hsa-miR-3692-5p**	1.95E-04	1.23E-03	-3.46	**hsa-miR-338-3p**	5.04E-12	8.96E-11	-1.59
**hsa-miR-4521**	6.20E-66	5.50E-64	-3.26	**hsa-miR-148a-5p**	5.86E-05	4.07E-04	-1.59
**hsa-miR-6832-3p**	8.70E-17	2.49E-15	-3.19	**hsa-miR-19a-3p**	1.67E-04	1.08E-03	-1.59
**hsa-miR-4481**	3.45E-02	1.15E-01	-3.13	**hsa-miR-15a-5p**	3.75E-21	1.45E-19	-1.57
**hsa-miR-5089-5p**	3.78E-02	1.24E-01	-3.00	**hsa-miR-1277-5p**	8.45E-10	1.17E-08	-1.54
**hsa-miR-4668-5p**	8.12E-09	1.03E-07	-2.97	**hsa-let-7a-3p**	3.20E-07	3.26E-06	-1.54
**hsa-miR-3140-5p**	3.32E-02	1.13E-01	-2.87	**hsa-miR-301a-5p**	1.20E-08	1.48E-07	-1.53
**hsa-miR-345-3p**	4.62E-08	5.26E-07	-2.82	**hsa-miR-551a**	5.21E-17	1.59E-15	-1.51
**hsa-miR-4448**	4.81E-10	7.11E-09	-2.76	**hsa-miR-7977**	9.55E-14	2.00E-12	-1.51
**hsa-miR-4466**	3.76E-02	1.23E-01	-2.69	**hsa-miR-4301**	6.71E-08	7.45E-07	-1.51
**hsa-miR-378f**	1.02E-02	4.23E-02	-2.64	**hsa-miR-548an**	4.18E-02	1.34E-01	-2.25
**hsa-miR-7703**	4.14E-02	1.34E-01	-2.47	**hsa-miR-545-3p**	1.55E-06	1.39E-05	-2.21
**hsa-miR-128-1-5p**	1.32E-05	1.06E-04	-2.36	**hsa-miR-16-2-3p**	7.02E-10	1.01E-08	-2.16
**hsa-miR-6750-3p**	7.12E-13	1.40E-11	-2.35	**hsa-miR-301b-5p**	3.73E-03	1.80E-02	-2.07
**hsa-miR-188-3p**	2.18E-03	1.11E-02	-2.30	**hsa-miR-4259**	1.13E-02	4.59E-02	-1.99
**hsa-miR-3916**	3.59E-02	1.19E-01	-2.29	**hsa-miR-1277-3p**	2.11E-04	1.30E-03	-1.90
**hsa-miR-16-1-3p**	2.58E-14	5.88E-13	-2.28	**hsa-miR-4531**	1.45E-03	7.78E-03	-2.26

Supplemental Table S7. miRNAs significantly increased in abundance (>1.5-fold) in hypoxia.

microRNA ID	p-value	FDR	Fold change (H vs. N)	microRNA ID	p-value	FDR	Fold change (H vs. N)
**hsa-miR-210-3p**	7.15E-105	1.06E-102	3.47	**hsa-miR-21-5p**	7.19E-07	6.86E-06	1.63
**hsa-miR-143-3p**	5.11E-90	6.48E-88	2.41	**hsa-miR-181b-5p**	6.75E-06	5.71E-05	1.60
**hsa-miR-483-5p**	1.31E-67	1.29E-65	2.43	**hsa-miR-30b-3p**	2.60E-05	2.01E-04	2.09
**hsa-miR-21-3p**	6.51E-46	5.25E-44	1.93	**hsa-miR-138-2-3p**	2.63E-05	2.01E-04	1.69
**hsa-miR-1275**	9.28E-45	6.87E-43	2.82	**hsa-miR-491-5p**	9.21E-05	6.20E-04	1.51
**hsa-miR-320b**	1.28E-35	8.77E-34	1.91	**hsa-miR-100-5p**	2.70E-04	1.63E-03	1.53
**hsa-miR-320c**	3.56E-33	2.11E-31	1.96	**hsa-miR-2115-3p**	3.49E-04	2.07E-03	3.74
**hsa-miR-138-5p**	3.27E-29	1.82E-27	1.94	**hsa-miR-342-5p**	3.86E-04	2.27E-03	1.56
**hsa-miR-483-3p**	5.63E-29	2.78E-27	1.89	**hsa-let-7d-3p**	4.43E-04	2.57E-03	1.69
**hsa-miR-744-5p**	1.55E-28	7.26E-27	1.92	**hsa-miR-4653-3p**	1.02E-03	5.67E-03	2.30
**hsa-miR-331-3p**	7.98E-24	3.37E-22	1.69	**hsa-miR-125a-3p**	1.26E-03	6.84E-03	1.60
**hsa-miR-30d-5p**	3.01E-20	1.11E-18	1.67	**hsa-miR-328-3p**	1.28E-03	6.94E-03	1.58
**hsa-miR-139-5p**	6.10E-19	2.01E-17	6.73	**hsa-miR-664b-3p**	2.07E-03	1.09E-02	1.78
**hsa-miR-197-3p**	1.19E-17	3.77E-16	1.88	**hsa-miR-3940-3p**	3.19E-03	1.56E-02	2.38
**hsa-miR-423-3p**	7.77E-17	2.30E-15	1.70	**hsa-miR-122b-5p**	4.07E-03	1.93E-02	1.61
**hsa-miR-27a-5p**	1.46E-16	4.06E-15	2.66	**hsa-miR-203a-3p**	5.37E-03	2.48E-02	3.84
**hsa-miR-145-5p**	1.66E-16	4.48E-15	1.66	**hsa-miR-766-3p**	7.48E-03	3.22E-02	2.09
**hsa-miR-181c-3p**	1.73E-16	4.53E-15	1.81	**hsa-miR-3187-3p**	7.65E-03	3.28E-02	1.53
**hsa-miR-143-5p**	4.12E-15	1.02E-13	1.92	**hsa-miR-3138**	9.02E-03	3.83E-02	2.15
**hsa-miR-1307-3p**	9.35E-15	2.24E-13	1.69	**hsa-miR-379-5p**	9.73E-03	4.09E-02	1.55
**hsa-miR-181d-5p**	8.26E-14	1.83E-12	1.69	**hsa-miR-664b-5p**	1.09E-02	4.49E-02	3.52
**hsa-miR-365a-5p**	9.88E-13	1.91E-11	4.11	**hsa-miR-3909**	1.11E-02	4.54E-02	1.74
**hsa-miR-652-3p**	5.03E-12	8.96E-11	2.18	**hsa-miR-3180-3p**	1.39E-02	5.58E-02	2.90
**hsa-miR-941**	7.13E-12	1.24E-10	1.65	**hsa-miR-6131**	1.49E-02	5.90E-02	2.72
**hsa-miR-320a-3p**	4.18E-11	7.01E-10	2.01	**hsa-miR-6511a-3p**	1.73E-02	6.66E-02	1.53
**hsa-miR-106b-3p**	1.34E-10	2.13E-09	1.62	**hsa-miR-4676-5p**	1.75E-02	6.72E-02	1.92
**hsa-miR-193b-5p**	1.53E-10	2.38E-09	3.43	**hsa-miR-219b-5p**	1.91E-02	7.23E-02	1.55
**hsa-miR-671-5p**	4.14E-10	6.34E-09	1.59	**hsa-miR-2277-3p**	1.95E-02	7.31E-02	1.94
**hsa-miR-330-3p**	4.69E-10	7.06E-09	1.77	**hsa-miR-877-3p**	2.11E-02	7.79E-02	1.73
**hsa-miR-1180-3p**	7.71E-10	1.09E-08	1.59	**hsa-miR-205-5p**	2.17E-02	7.95E-02	8.46
**hsa-miR-320d**	1.93E-09	2.62E-08	2.02	**hsa-miR-509-3p**	2.47E-02	8.93E-02	2.70
**hsa-miR-4791**	1.99E-09	2.63E-08	1.51	**hsa-miR-760**	2.52E-02	9.06E-02	1.73
**hsa-miR-877-5p**	7.89E-09	1.01E-07	1.59	**hsa-miR-6787-3p**	3.04E-02	1.06E-01	2.80
**hsa-miR-193b-3p**	1.25E-08	1.52E-07	1.93	**hsa-miR-2277-5p**	3.06E-02	1.06E-01	1.52
**hsa-miR-132-3p**	2.61E-08	3.05E-07	1.66	**hsa-miR-3679-5p**	3.44E-02	1.15E-01	1.67
**hsa-miR-200b-5p**	2.54E-07	2.65E-06	1.62	**hsa-miR-212-3p**	4.79E-02	1.52E-01	1.66
**hsa-miR-92b-3p**	5.21E-07	5.14E-06	1.56	**hsa-miR-654-3p**	4.86E-02	1.54E-01	1.61

			3.47	**hsa-miR-224-3p**	4.97E-02	1.57E-01	2.02

Supplemental Table S8. miRNA-mRNA interaction list with up-regulated miRNA and down-regulated mRNA targets.

Supplemental Table S9. miRNA-mRNA interaction list with down-regulated miRNA and up-regulated mRNA targets.

Supplemental Table S10. Read count, alignment and conditions for RNA sequencing
